# Activity and Diversity of Microorganisms in Root Zone of Plant Species Spontaneously Inhabiting Smelter Waste Piles

**DOI:** 10.3390/molecules25235638

**Published:** 2020-11-30

**Authors:** Sylwia Siebielec, Grzegorz Siebielec, Piotr Sugier, Małgorzata Woźniak, Jarosław Grządziel, Anna Gałązka, Tomasz Stuczyński

**Affiliations:** 1 Department of Microbiology, Institute of Soil Science and Plant Cultivation—State Research Institute, Czartoryskich 8, 24-100 Pulawy, Poland; M.Wozniak@iung.pulawy.pl (M.W.); jgrzadziel@iung.pulawy.pl (J.G.); agalazka@iung.pulawy.pl (A.G.); 2 Department of Soil Science Erosion and Land Protection, Institute of Soil Science and Plant Cultivation—State Research Institute, Czartoryskich 8, 24-100 Pulawy, Poland; gs@iung.pulawy.pl; 3 Department of Botany, Mycology and Ecology, Institute of Biological Sciences, Maria Curie-Skłodowska University, Akademicka 19, 20-033 Lublin, Poland; piotr.sugier@poczta.umcs.lublin.pl; 4 Faculty of Science and Health, The John Paul II Catholic University of Lublin, Konstantynów 1 H, 20-708 Lublin, Poland; Tomasz.Stuczynski@sgs.com

**Keywords:** functional diversity, genetic diversity, metal mobility, ecotoxicity

## Abstract

The aim was to assess plant driven changes in the activity and diversity of microorganisms in the top layer of the zinc and lead smelter waste piles. The study sites comprised two types (flotation waste—FW and slag waste—SW) of smelter waste deposits in Piekary Slaskie, Poland. Cadmium, zinc, lead, and arsenic contents in these technosols were extremely high. The root zone of 8 spontaneous plant species (FW—*Thymus serpyllum*, *Silene vulgaris*, *Solidago virgaurea*, *Echium vulgare*, and *Rumex acetosa*; and SW—*Verbascum thapsus*; *Solidago gigantea*, *Eupatorium cannabinum*) and barren areas of each waste deposit were sampled. We observed a significant difference in microbial characteristics attributed to different plant species. The enzymatic activity was mostly driven by plant-microbial interactions and it was significantly greater in soil affected by plants than in bulk soil. Furthermore, as it was revealed by BIOLOG Ecoplate analysis, microorganisms inhabiting barren areas of the waste piles rely on significantly different sources of carbon than those found in the zone affected by spontaneous plants. Among phyla, Actinobacteriota were the most abundant, contributing to at least 25% of the total abundance. Bacteria belonging to *Blastococcus* genera were the most abundant with the substantial contribution of *Nocardioides* and *Pseudonocardia*, especially in the root zone. The contribution of unclassified bacteria was high—up to 38% of the total abundance. This demonstrates the unique character of bacterial communities in the smelter waste.

## 1. Introduction

The mining and smelting of zinc (Zn) and lead (Pb) has contributed to the creation of a large number of solid waste deposit sites as its legacy. The sites constitute a real threat not only for the environment but also for human health, due to extremely high concentrations of potentially toxic trace elements (PTTE), uncontrolled fugitive dust movement in the air and exposures, and the runoff and leaching of contaminants such as zinc, lead, cadmium (Cd), or arsenic (As). Lack of vegetation induces the secondary dispersion of metals to adjacent soils, water bodies, and living organisms. In most cases, the presence of hazardous elements causes that the sites are not suitable for urban redevelopment, and they require land reclamation to reduce their impact on human health and the ecosystems [1].

According to the data collected through the European Environment Information and Observation Network for Soil (EIONET-SOIL), there are 2.5 million potentially contaminated sites caused by point pollution, and 342,000 identified contaminated sites [2]. In many regions, such as Silesia in Southern Poland, Zn and Pb slag waste piles are distributed across a mosaic of various land use types, including urbanised areas, arable lands, hobby gardens, forests, and recreational areas. In the Silesian Voivodship, metalliferous waste heaps are spread over an area of about 160 km^2^ [1]. These waste piles are often characterised by harsh physical and water conditions, which result in extremely unfavourable conditions for plant growth due to a lack of nutrients, high concentration of phytotoxic metals, and limited access to water. The lack of organic matter in these industrial soils causes very low water retention, which makes the growth of plants very difficult [3]. The secondary dispersion of PTTEs from smelter waste piles causes further pollution of adjacent soils, which are often used as arable lands or for hobby gardening [3].

Stuczynski et al. [3] and Siebielec et al. [4] have described the effectiveness of biosolids and limestone application in smelter waste pile reclamation in the Upper Silesia. The long term evaluation of pilot reclamation sites indicates that the tested technology ensured a fairly compact plant cover in landfill sites, limiting the secondary spread of pollutants.

Processes of spontaneous vegetation in toxic zinc (Zn) and lead (Pb) smelter waste piles are very slow; however, when resources for waste pile reclamation and interest in reclamation actions are limited, they might play an important role in reducing the environmental and health risk in the long term. The annual management cost of contaminated sites in Europe is estimated to be around 6 billion Euro [2], and therefore many toxic waste deposits are not approached for reclamation, especially in countries without efficient strategies for site management and the funding of reclamation actions. 

Permanent plant cover, which effectively limits the dispersion of metals, might be based on both introduced grasses and spontaneous species to ensure greater plant diversity and the resistance of such an ecosystem [4]. Wójcik et al. [5] provided a list of the most common spontaneous plant species growing on flotation and slag waste piles in Piekary Slaskie in Upper Silesia, Poland. It can be assumed that microorganisms contribute to creating conditions for plant growth through processes that limit metal toxicity, retain water in the upper layer, and provide nutrients. In our own research, we have assessed the activity of microorganisms in the upper layer of the smelter pile, and plant diversity after 19 years of land reclamation through treatment with biosolids and lime. The treatment involved various rates of biosolids and by-product lime, and their combinations [4]. We concluded that high plant species diversity allows greater plant cover resistance to stress conditions caused by metal toxicity and other constrains, such as low water holding capacity and low nutrient availability [4].

Most Zn and Pb waste deposits are almost barren, but sparse spontaneous vegetation provides valuable information on interaction between plants and microorganisms in the root zone. The growing conditions for both plants and microorganisms in such constructed soil are extremely harsh, due to the high content of heavy metals and deficit of nutrients and water. So far, there is no sufficient data available on the role of microorganisms in the colonisation of waste piles by spontaneous vegetation, and through which microorganisms promote plant growth in such toxic systems. The aim of the work was therefore to compare the activity and diversity of microorganisms in the root zone of spontaneous plants, and from the barren areas of the smelter waste piles. The top layer of the studied waste piles will be referred to as industrial soil throughout the article. Such information will shed light on interactions between microorganisms and the most frequent spontaneous plant species inhabiting the two types of waste deposits in Piekary Slaskie, Poland.

## 2. Results and Discussion

### 2.1. Soil Chemical Characteristics

The chemical and physical characteristics of the investigated root zone soils are presented in Table 1. Both waste types were alkaline: the pH of flotation waste (FW) soil ranged from 8.00 to 8.23, and the pH of slag waste (SW) from 8.23 to 8.53. The soils are characterized by extremely high salinity as demonstrated by EC (electrical conductivity) measurements—the EC values ranged from 116 to 388 mS·m^−1^. EC values for arable lands in Poland do not exceed 30 mS·m^−1^, as documented within the national monitoring of soil quality [6]. The content of organic matter (OM) in the waste was relatively high as was earlier discovered by Siebielec et al. [4]. OM in these wastes apparently originates from residual elemental carbon from the smelting process [3]. It can be assumed that elemental carbon as coke fuel residue in the slag waste does not have properties and functions similar to organic matter in agricultural soils. It probably also does not considerably increase the water retention capacity of the soils, nor serve as a carbon source for the soil microorganisms. Generally, *S. gigantea* grew on sites (SW) with lower OM content than other species. The greatest OM level was found in the *V. thapsus* root zone.

Phosphorus (P) extractability was very low in all sampling locations (Table 1); however the available phosphorus was greater in all root zone soils than in control soils, which might indicate the positive effect of plant/microorganism interaction on P availability to plants. It is worth noting that average available P in arable soils in Poland is almost 10 times greater (167 mg P_2_O_5_ kg^−1^) [6]. In such P-deficient conditions as observed on FW and SW smelter waste heaps, the microbially induced P solubilisation processes might be critical for plant growth. In pilot reclamation of slag waste deposit, the P deficit was overcome by using P-rich municipal sewage sludge [3].

Potassium (K) extractability, which simulates its availability to plants, was rather low in control soils but not extremely low (Table 1). Soils affected by plants had much greater K extractability than barren soils, except for *E. vulgare*, *R. acetosa*, and *E. cannabinum*. The mechanism of K solubilisation in the root zone of several plants is not clear; however, recent studies have documented isolation of K-solubilising bacteria both from K minerals and rhizosphere of cropped plants [7].

Ammonia nitrogen (N-NH_4_) was substantially greater in all soils collected below plants than from barren soil—in FW from 1.7 to 4.8-fold greater (Table 1). In SW the differences were lower, but the trend remained the same. N-NH_4_ production was most stimulated in root zone of *S. virgaurea* and *T. serpyllum*, suggesting relatively high activity of microorganisms involved in transformation of organic nitrogen compounds.

The nitrate content did not significantly differ between sampling locations ranging from 2.53 to 6.63 and from 2.39 to 8.71 mg N-NO_3_ kg^−1^, in FW and SW, respectively. N cycling in the SW soils was slightly different from in the FW, since nitrates dominated over ammonia content in the SW.

As expected on the basis of previous studies [4,5], the metal content in the industrial soils was extremely high for Cd, Zn, Pb, and As (Table 2). FW was extremely rich in Zn (85,500–112,200 mg kg^−1^), the content of which was very uniform, and no significant differences were found between particular sampling locations. This means that Zn constitutes approximately 10 percent of the FW. Despite this Zn content, some wild plant species were able to grow on soils formed on such waste deposits. The content of Pb in FW reached a level of 18,550–24,130 mg kg^−1^ across the entire site. Arsenic content was also very high, ranging from 2090 to 2560 mg kg^−1^. The total cadmium content in FW did not show great variability, at the level of 418–556 mg kg^−1^, and its contents in bulk and root zone soils were similar. The copper content in FW is low (24–51 mg kg^−1^), at a level only slightly greater than commonly found in Polish agricultural soil [6]. Interestingly, plants were growing in locations with greater total K content than in the control soil. The FW soil was relatively rich in calcium (Ca)—up to 52,560 mg kg^−1^, and magnesium (Mg)—up to 26,990 mg kg^−1^. Iron (Fe) is a major component of the FW, and ranged from 25.5% to 32.5%.

The SW area was characterised by lower Zn content (up to 32,560 mg kg^−1^) than in FW, but was still at extreme levels (Table 2). Pb, Cd, and As were at very high levels in FW, in a barren area at a level comparable to that in FW. It must be noted that the plants grew in locations with significantly lower contents of Zn, Cd, and Pb, which indicates that plant appearance might be driven by the toxicity of metals, especially Zn and Cd. The major difference between the waste types is the high content of copper (Cu) and much greater Ca content in SW than in FW (12.8% and 5.2% Ca in control samples, respectively). Very high Fe content in both wastes apparently contributes to limiting metal mobility and phytotoxicity [8]. 

The major phytotoxicity factor in such waste piles is the amount of plant available Zn [3]. Toxic levels of Zn were confirmed by the Ca-chloride extraction (Table S1, Supplementary Materials). Extractable Zn was definitely higher in FW than in SW, indicating a greater risk of secondary Zn release from the waste through leaching and/or run-off. The analysis revealed more intense processes of Zn, Cd, and Pb solubilisation in the root zone of all plants growing on FW, as opposed to SW where metal extractability was generally lower in soils affected by plants.

### 2.2. Plant Chemical Composition

The chemical composition of the investigated plants is presented in Table 3. A high variability in metal content was observed. For example, Zn content ranged from 236 mg kg^−1^ in *S. gigantea* to 20,870 mg kg^−1^ in *E. vulgare* (Table 3). Such large variability suggests different plant physiological strategies regarding adaptation to extreme metal contents in soil: avoidance, inactivation, and accumulation [5]. The lowest Zn content was recorded for *S. gigantea* and *E. cannabinum*, at levels which are considered as non-toxic even for crops. This level is specified as >300 mg kg^−1^ of dry matter [9]. Both species were collected from SW. Such a low uptake of Zn by these two species is linked to much lower Zn extractability in the SW soil. Zn in *S. virgaurea* growing on FW, and *V. thapsus* collected from SW was only slightly higher. Conversely, *S. vulgaris*, *R. acetosa*, and *T. serpyllum* accumulated from 2847 to 6337 mg Zn in a kilogram of dry matter. This indicates the strong resistance of these plants to enhanced Zn uptake. Finally, *E. vulgare* accumulated 20,870 mg Zn kg^−1^ (above 2% of dry matter) when growing on FW. Such concentration has been rarely found in plants classified as hyperaccumulators [10]. Separate studies would be needed to explain such high uptake of Zn by E. vulgare grown on FW. However, some authors have pointed out increased Zn uptake under stress conditions related to low nutrients [11], also observed in our study (Table 1). Cd and Pb content in plants in our study exhibited similar pattern as Zn (Table 3). *E. vulgare* accumulated 4351 mg Pb kg^−1^ (0.43% of dry matter), which is also a rare level. It is worth noting that this plant also contained high levels of Fe and Ca, which play a role in the inactivation of trace metals in plants. Sarret et al. [12] reported the production of Zn-containing biogenic calcite through the trichomes as a mechanism involved in Zn detoxification in plant cells. Shanmugam et al. [13] reported Fe-mediated Zn tolerance in Arabidopsis, attributing its major mechanism to increased glutathione accumulation. *E. vulgare* exhibited the greatest bioconcentration factor (BF) for Zn, Cd, Pb, and As, followed by *T. serpyllum* (Table 3).

Stefanowicz et al. [14] provided data on the accumulation of elements in plants spontaneously populating metallurgical waste dumps. Plants growing in heaps accumulated excessive amounts of some elements in tissues, on average 1.3–52 mg Cd kg^−1^, 9.4–254 mg Pb kg^−1^, and 134–1479 mg Zn kg^−1^. The highest concentrations of Cd, Pb, and Zn were found in the roots of *Euphorbia cyparissias* L., *Fragaria vesca* L., and *Potentilla arenaria* Borkh. Plant roots can take much more Pb than leaves. The translocation of this element to roots might constitute as much as 95% of the total uptake. Roots take lead rather easily, but it is transported to the above-ground parts in limited quantities [15]. 

Wójcik et al. [5] conducted research involving an extensive collection of spontaneous plants growing on various smelter waste piles, including the two covered by our study, followed by an analysis of the accumulation of metals in plant tissues. The highest average concentrations of Zn, Pb, and Cd were found in *Anthyllis vulneraria* L. (902 mg kg^−1^), *E. vulgare* L. (117 mg kg^−1^), and *Hieracium piloselloides* Vill. (26.9 mg kg^−1^), respectively. In their study, metal accumulation was quite diversified, depending on the individual plant. As in our study, the concentrations were much higher in *E. vulgare* grown on the flotation waste—the highest Zn content was 1173 mg kg^−1^. Our data indicate that even species recommended to use in reclamation must be carefully tested, since in toxic environments such as SW, they can accumulate extremely high amounts of metals with an impact on the metal transfer in a food chain of the reclaimed ecosystem. Species posing a considerable risk for Zn, Cd, and Pb accumulation include *E. vulgare* in particular, and to a lesser extent *T. serpyllum* and *R. acetosa*.

### 2.3. Soil Enzyme Activities

Soil microorganisms are key elements in the functioning of the terrestrial environment. They take part in the process of nutrient cycling, facilitating plant growth as well as enhancing phytoremediation processes [16,17]. Chemical stress caused by high concentration of heavy metals affects the number and metabolic activity of microorganisms in soils. High pollution with heavy metals in soils can decrease microbial diversity or disturb functional activity, which may affect nutrient availability for plants [17,18,19]. However, microorganisms that occur in contaminated soils demonstrate the ability to survive in toxic environments through the development of various adaptive mechanisms [20,21].

Relatively high enzymatic activity of dehydrogenases and both acid and alkaline phosphatase was found in soils from root zone of *T. serpyllum*, *S. vulgaris*, and *S. virgaurea*, compared to control areas (of both FW and SW) (Table 4). Soils collected from individual plants of the same species also showed quite pronounced differences in the activity of the enzymes. Since there was no high diversity of metal contents, soil pH and OM within one heap (Table 1 and Table 2, Table S1), we assume that the significant differences in the enzymatic activity were mostly stimulated and driven by plant-microbial interactions. Similarly, Kim et al. [22] observed activity of dehydrogenases driven by vegetation in abandoned mine area soil, highly contaminated with As. Stimulation of soil enzymes by root exudates in Cu contaminated soil has been documented by Vogeler et al. [23].

### 2.4. Abundance of Cultivable Microorganisms

The classical plate dilution methods have limitations related to their low resolution and the fact that they characterize only a cultivable part of microbial community. However, they still provide insight into abundance of certain groups of microorganisms. In contrast to the dehydrogenases activity, the count of culturable soil bacteria was in general greater in root zone soils of SW. It is possible that they were relatively abundant, but less active. The plants definitely stimulated the number of bacteria, as shown in Table 5. The count of culturable soil fungi was even more highly dependent on interaction with plants. FW was especially indigent in fungi in the barren area.

The highest number of bacteria was recorded for *S. gigantea*, which grew on SW, but the root zone of this plant was not rich in fungi. The opposite relationship was observed for *S. vulgaris* (Table 6). The other plants did not exhibit such discrepancies. 

Ammonification is a microbially driven process in which amino groups (NH_2_) associated with organic forms of nitrogen are converted into ammonia (NH_3_) or ammonium (NH_4_^+^). Ammonium is then available for plants or becomes a subject of the nitrification processes. The highest number of culturable ammonification bacteria was found in the root zone of *S. gigantea* (SW) and *R. acetosa* (FW). Interestingly, FW was poor in ammonification bacteria except for the root zone of *R. acetosa*. In most root zone soils of FW, it was much lower than in barren SW.

The bacteria growing on the media specific to *Azotobacter*, which is responsible for N-fixing from air, were not found in any sample, regardless of the type of waste and presence of plants, similarly as observed by Siebielec et al. [4] on the reclaimed slag in Piekary, where only a combination of sludge and lime soil amendments enabled *Azotobacter* growth.

Oligotrophs have the ability to grow under low substrate concentrations (e.g., carbon), and therefore in general exhibit a higher substrate utilisation efficiency. The *Acidobacteria* and *Verrucomicrobia* are among the phyla consistently associated with oligotrophic environments [24]. Copiotrophic bacteria tend to grow in highly organic substrate conditions and therefore are more susceptible to low availability of carbon sources. In all our samples, the count of oligotrophs was slightly greater than the number of copiotrophic bacteria, including FW and SW controls. The greatest difference between oligotrophs and copiotrophs (>2-fold greater number of oligotrophs) was observed in the root zone of S. vulgaris and S. gigantea, where the OM contents were the lowest. The total OM content, as measured by loss on ignition, is rather high in all samples, but the carbon availability for bacteria is apparently limited. Regardless of the harsh conditions, oligotrophic bacteria are in general in balance with more carbon dependent copiotrophs—this indicates potential mechanism of microbial and plant species survival and adaptation through broadening functional diversity of the microbiome.

### 2.5. Effects of Soil Chemistry on Abundance and Activity of Microorganisms

Regression models were generated using stepwise technique to identify combinations of soil parameters responsible for soil microbial abundance or enzyme activities in soils collected from root zone of spontaneous plants (Table 6). Among parameters characterizing abundance of culturable microorganisms, high R^2^ was obtained for count of oligotrophic bacteria and ammonification bacteria (0.84 and 0.85, respectively). Interestingly, in the case of total number of culturable bacteria soil, chemical variables were responsible for only 33% of its variability. Apparently, 2/3 of variability can be mostly attributed to the interaction of bacteria with various spontaneous plants. In general, total contents of Ca, Mg, K, Fe, or Cu went into models. Among extractable contaminants, only As appeared in the regression models for total abundance of bacteria, oligotrophs, copiotrophs, and ammonification bacteria with negative coefficients, suggesting a detrimental effect of As on bacteria (Table 6).

In the case of enzyme activities, it seems that the effect of soil chemical characteristics was stronger—they explained 86–89% of the variability across sampling locations. Not only total contents of elements went into models, but also content of N-NH_4_, salinity (EC) and OM (Table 6). In the regression model for acidic phosphatase, OM had a negative coefficient so that its greater content meant lower enzyme activity. There was a positive relationship between content of ammonia and both phosphatases and dehydrogenases, which might indicate that greater ammonia availability resulted from plant driven activity of microorganisms [22]. There was one parameter representing solubility of toxic contaminants in the model developed for activity of dehydrogenases—extractable Cd, but with positive value. Literature does not provide any information on relationships between chemical status and abundance or activity of microorganisms in such spontaneously vegetated waste piles. In papers focused on soil, most authors report negative effects of long-term exposure to metals on microbial activity or describe shifts in microbial community structure [25,26]. However, there are also contradictory reports [27]. Stuczynski et al. [3] developed regression models for enzyme activities in the waste pile reclaimed with biosolids and by-product lime. In their study, little variability in enzyme activities was assigned to soluble metals—they were mostly dependent on content of organic matter, introduced with substantial amounts of biosolids, and availability of nutrients such as P and K.

Interestingly, soil pH did not enter any regression model (Table 6). Correlation analysis revealed that there was no significant relationship between pH and abundance of any measured group of culturable microorganisms, but a negative relationship with enzyme activities was observed with r in the range −0.59 to −0.76 (Table S2). Weak effects of pH on microbial parameters might have been related to the observed narrow range of pH (8.0–8.5). As documented in previous studies, pH of smelter wastes disposed in the region did not exhibit large variability, remaining in a range from neutral to alkaline [5]. OM content alone was not significantly correlated to any of microbial parameters.

Among variables characterizing solubility of potentially toxic elements, extractable As was strongly correlated with acidic phosphatase and less with other enzymes. This result and its appearance in the regression models suggest that as was most detrimental to microorganisms in the studied soil ecosystem among all contaminants tested.

### 2.6. Plant Driven Changes in Functional and Genetic Diversity of Microorganisms

Biolog EcoPlates is an effective method of evaluating changes in the metabolic potential of microbial communities. The utilisation dynamics of 31 specific carbon substrates on Biolog EcoPlates provides results for evaluating the effects of various drivers on soil microbial community [28,29]. 

**Figure 1:**
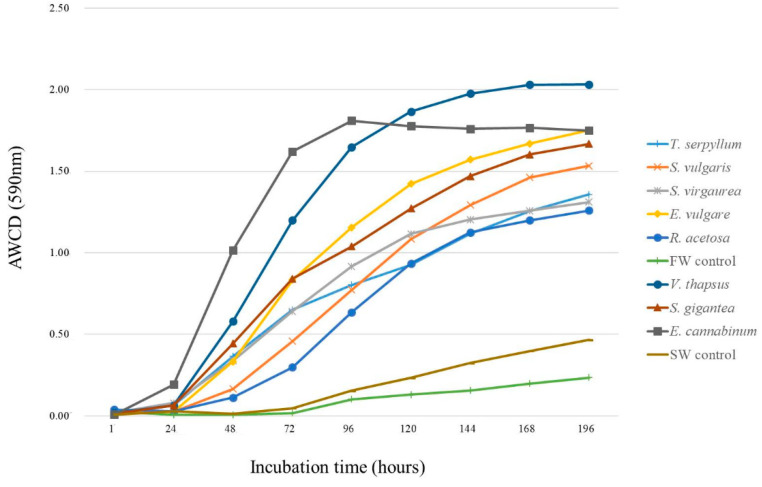
Changes in the average well colour development (AWCD) of carbon source utilisation in the Biolog EcoPlate from 1 h to 196 h (values are means, *n* = 3).

Biolog Ecoplates were read between the 0 and 196 h in order to determine the relationship between the utilisation of various carbon sources and time. The average well colour development (AWCD) dynamics are presented in Figure 1. The value of AWCD indicates the general metabolic activity of the microorganisms in a given sample. As shown in Figure 1, the AWCD value of all microbial communities in root zone increased remarkably over the period of incubation. The results demonstrated that the AWCD of all soil samples showed a visible lag phase within the first 24 h (except for soil representing *E. cannabium* grown on FW). The AWCD then increased sharply, presenting a diversity of microbial communities from investigated soils. Microorganisms in the FW and SW control samples also showed increased activity after only 72 h incubation, and the increase was much less sharp than in the root zone samples. The AWCD values for all incubation times of *V. thapsus* and *E. cannabinum* were higher among the eight root zone soils than the other samples. The time selected for deeper analysis of the BIOLOG EcoPlates data was 144 h. This seems to be the optimal time to analyse the data, since the AWCD showed a tendency to stabilise (Figure 1).

Table 7 shows the evaluation of the microbial functional diversity. The highest level of differentiation between the tested soil samples was recorded for the AWCD parameter. Among the samples selected from the FW, the statistically highest value of AWCD was attributed to *E. vulgare*. Soils representing *S. vulgaris*, *S. virgaurea*, and *R. acetosa* constituted one relatively homogeneous group. In general, SW samples were characterised by higher AWCD values than FW samples. The soil collected under *V. thapsus* (SW) exhibited the highest AWCD value (AWCD 590 nm = 1.98). Control samples of both FW and SW showed the lowest AWCD values, which indicates that microorganisms from these barren spots had the lowest affinity to the individual carbon substrates. Other indicators did not show such a large difference. The number of carbon substrates used (R—richness index) was not different between various plant species, regardless of the type of smelter waste as parent material for industrial soils formed. The control samples showed the lowest R values. The R index in the FW control was also significantly lower than in the barren SW. A similar trend as in the case of R was observed for the H’ index. The Eveness index (J’) in this particular case characterises the distribution of carbon sources utilised by the bacteria. The index value was not different between plant species, but the values for control were much lower, exhibiting significantly less capacity to utilise various C substrates. 

The Biolog EcoPlate analysis demonstrated that the microbial communities from the tested samples had diverse ability to utilise the 31 carbon sources. The metabolic profile of the microbial community, as based on utilisation of the individual carbon substrates, was characterised using a cluster analysis and was visualised as a heatmap (Figure S1). The highest utilisation is shown in red, and the lowest consumption is in green. In the control samples collected from the SW barren areas, microorganisms did not use the carbon sources intensively, but utilised d-Xylose, Tween 80, and l-threonine preferentially. In the FW control samples, the utilisation of 2-hydroxy-benzoic acid and phenylethylamine dominated. In soil samples in the root zone characterised by the most intensive carbon utilisation, microbial communities transformed significantly different C sources. In *V. thapsus* (SW), the maximal use of the substrate was recorded for Putrescine, d-Glucosaminic acid, and gamma-Hydroxybutyric acid. Microorganisms representing *E. cannabinum* most intensively consumed 2-Hydroxy-Benzoic acid (salicylic acid—SA) and phenylethylamine as the only root zone community tested, but only mildly used putrescine and d-Glucosaminic acid (Figure S1). It has been shown that SA is a plant hormone that regulates various aspects of plant growth, flower induction, and ion uptake, and has signalling roles in disease resistance [30,31]. Apparently, *E. cannabinum* produces a substantial amount of SA, possibly to increase its resistance to harsh conditions on the SW soil. It is also possible that microorganisms in its rhizosphere have developed the ability to use this carbon source as an example of plant-microbial interactions, specific to this smelter waste pile. Microorganisms representing *E. vulgare* (FW), which were the most active in C transformation among the FW communities, intensively utilised Glucose-1-Phosphate, beta-Methyl-DGlucoside, and alpha-Cyclodextrin.

**Figure 2:**
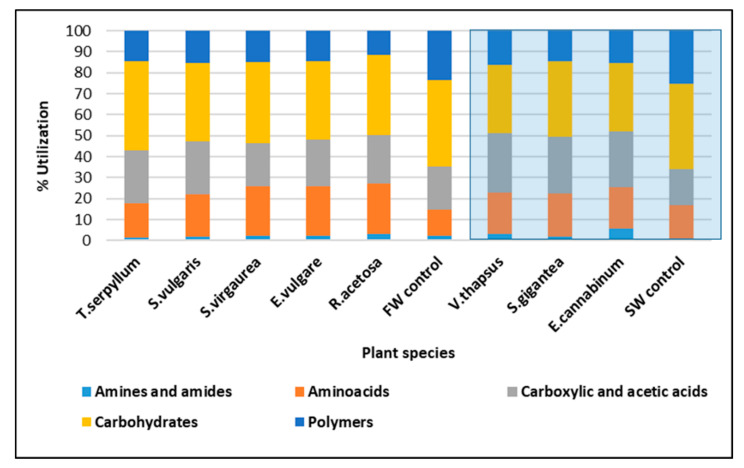
Average utilisation of groups of carbon substrates in FW and SW soils based on 144 h incubation (*n* = 3). White background: FW; blue background: SW.

The ability of microorganisms to utilise the C sources as combined within five major groups of C compounds, amines and amides, amino acids, carboxylic and acetic acids, carbohydrates and polymers, is shown in Figure 2, which demonstrates the high variability between microbial communities belonging to particular plant species. Amines and amides were consumed to the greatest extent (as measured by the percentage of all consumed C substrates) by communities of *E. cannabinum*. Amino acids constituted the largest percentage in C utilisation for microorganisms associated with *S. virgaurea*, *E. vulgare*, and *R. acetosa*. Interestingly, carbohydrates were most preferentially utilised by *T. serpyllum*, but also in both controls where microbial communities were not affected by plants. Furthermore, in the control samples of FW and SW soils, microorganisms were able to preferentially consume polymers, represented here by relatively complex C sources such as α-cyclodextrin, glycogen, Tween 40, and Tween 80 (Figure 2). This data clearly shows that microorganisms inhabiting barren areas of the waste piles rely on different proportion of carbon sources than those found under the spontaneous plants. The data also demonstrates the interactions between plants and root zone microorganisms leading to enhanced microbial activity and shifts in the ability to utilise various C substrates. We found that carbon source utilization indicator such as AWCD and BIOLOG-derived diversity indexes are good parameters to depict microbial functional diversity in initial soils formed on smelter slags. Our data indicate that particular plant species develop an individual pattern of microbial functionality, which is reflected in distinct profiles of carbon utilization sources. It is worth mentioning that *E. vulgare* represents the highest ability to utilize different carbon sources among all FW soils—interestingly, this coincides with the highest accumulation of zinc in plant biomass of above 2%.

**Figure 3:**
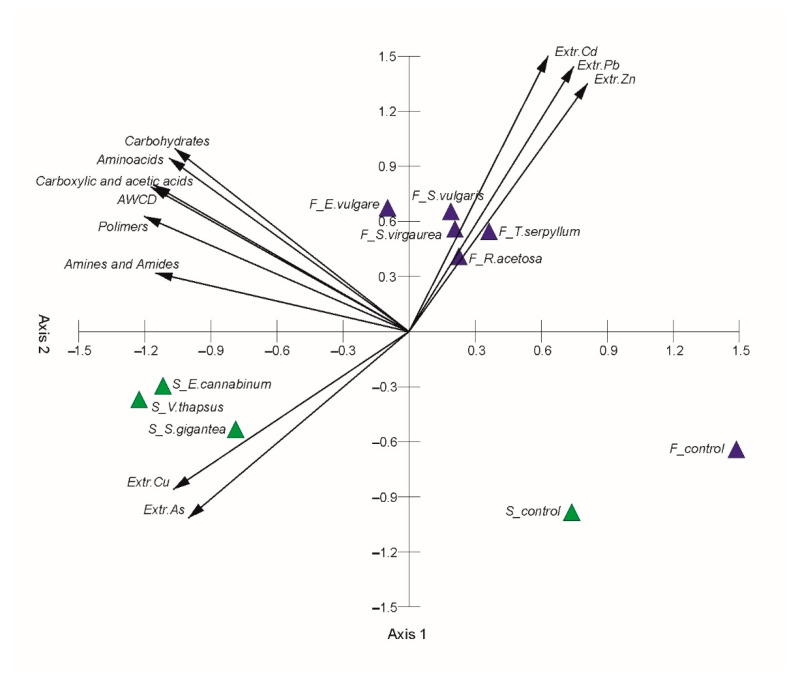
Principal component analysis (PCA) of metabolic activity parameters (based on EcoPlate) and extractable PTTE in FW and SW soils.

Based on principal component analysis (PCA) analysis, the tested soils were mutually positioned based on the similarity of patterns of utilisation of C substrates. The AWCD, since it was responsible for the greatest diversity between soils, the percentage utilisation of individual groups of carbon sources, and CaCl_2_-extractable PTTE were used for this analysis. The total variance explained by Axis 1 and Axis 2 was 94.69% (61.06 and 33.63%, respectively) (Table S3). Axis 1 shows a negative correlation with all metabolic activity parameters and extractable Cu and As in root zone soils, and a positive correlation with extractable Cd, Pb, and Zn (Figure 3, Table S3). Therefore, this principal component separates samples located on the left side of the ordination space (*E. cannabinum*, *V. thapsus*, *S. gigantea*) with high values of the carbohydrates, aminoacids, carboxylic and acetic acids, polimers, amines and amides, AWCD, and extractable Cu and As from the rest of the samples. Moreover, there is a positive correlation between extractable Cu and As in root zone soils and all metabolic activity parameters (Table S4). These relations can be of indirect character, resulting from the generally greater metabolic activity in SW. The other extractable trace elements (Pb, Zn) were not significantly correlated and in general exhibited negative coefficients of correlation. Axis 2 exhibits a high positive correlation with extractable Cd, Pb, and Zn, and negative correlation with extractable Cu and As (Figure 3, Table S3). This principal component separates samples situated in the upper part of the ordination space (*S. vulgaris*, *S. virgaurea*, *E. vulgare*, *T. serpyllum*, *R. acetoa*) from samples located in the lower part of the ordination space with higher content of Cu and As. A long distance between the bulk soils (controls) and the root zone soils document strong influence of plant species on microbial activity and diversity of carbon sources used. It is characteristic for both SW and FW industrial soils.

**Figure 4:**
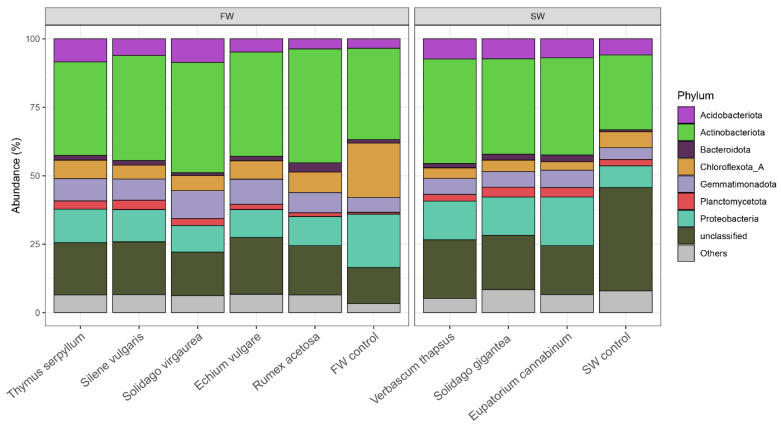
Most abundant phyla in the root zone of the spontaneous plant species.

The communities of the soil samples were dominated by seven phyla: Acidobacteriota, Actinobacteriota, Bacteroidota, Chloroflexota, Gemmatimonadota, Planctomycetota, and Proteobacteria (Figure 4). Actinobacteriota were the most abundant, contributing to at least 25% of the total abundance. In soil, they behave much like fungi, helping to decompose the organic matter of dead organisms so the molecules can be taken up by plants. Gemmatimonadetes have been found in a variety of arid soils [32]. Planctomycetes are a phylum of aquatic bacteria. Other phyla contributed to less than 10% of the total abundance of bacteria. The contribution of Actinobacteriota in both control samples was always lower than in root zone soils, regardless of the plant species.

Chloroflexota and Proteobacteria made a much greater contribution in barren FW than in the FW root zone soils (Figure 4). Interestingly, the contribution of unclassified bacteria was relatively high, ranging from 13% to 23% in all root zone samples and FW control. The exception was SW control where it reached 38% of the total relative abundance. This demonstrates the unique character of bacterial communities in the slag smelter waste. The invasion of spontaneous plants into the waste piles apparently converts the slag waste communities into a more soil-like structure of bacterial communities; however, the substantial contribution of Planctomycetota is a rather unexpected observation. 

**Figure 5:**
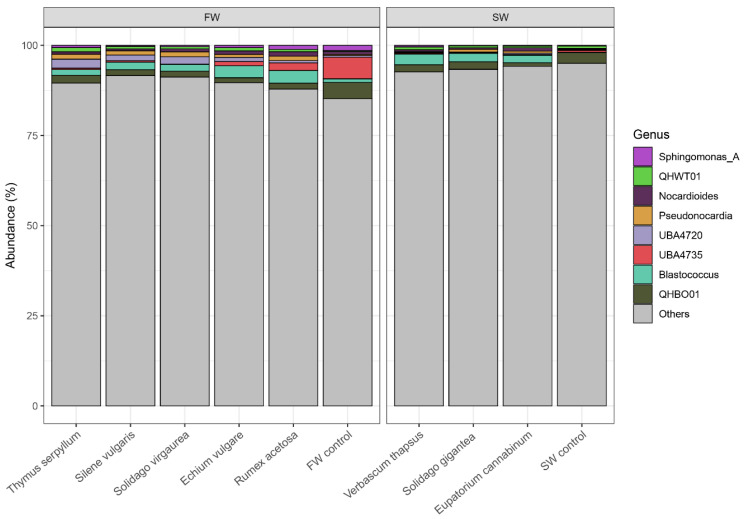
Most abundant genus in the root zone of the spontaneous plant species.

Figure 5 presents percentage contribution of 8 most prevalent genera to the total bacterial abundance in root zone of plant species, as compared to bulk soils. They constituted up to 15% of the total abundance. The other genera contributed to at least 75% of total abundance, but their individual contribution was not that significant as in the case of these 8 most prevalent ones. *Blastococcus* was the most representative genus in the root zone of plants growing on SW (over 2% abundance), followed by the not fully recognised QHBO01 (Figure 5). This genus was dominant in the SW control, whereas the contribution of *Blastococcus* was drastically lower. *Blastococcus* strains have been reported as adapting to drought conditions [33]. Touceda-González et al. [34] reported a high abundance of *Blastococcus* genera in the rhizosphere of *Alyssum serpyllifolium*—nickel hyperaccumulating plant species growing in ultramafic soils. The metal resistance of *Blastococcus* was confirmed by Gtari et al. [35]. Our study confirmed the ability of this genera to adapt to extreme conditions and its interaction with ornamental plants. *Blastococcus* was most abundant among all genus in the rhizosphere of *R. acetosa*, *E. vulgare*, and *S. vulgaris*, and although it was not dominant in other plants, it was still well represented.

The analysis also revealed the substantial contribution of *Nocardioides* and *Pseudonocardia* genera, especially in root zone. The root zone of *T. serpyllum*, *S. virgaurea* was dominated by the unrecognised genera UBA4720. *Nocardioides* are known for salt tolerance and the release of phosphatases [36]. These both abilities might have played a role in plant-microbial interactions observed in FW and SW wastes. *Nocardioides nitrophenolicus* was first isolated from industrial wastewater [37]. Essel et al. [38] found *Nocardioides* in cultivated soil under cereal/legume rotation with a larger contribution in the root zone than in bulk soil. A similar situation was observed in our study—the genus existed in bulk samples (FW and SW controls) but spontaneous plants induced its greater contribution to the total bacterial community.

Some species of the actinomycete *Pseudonocardia* are known for their ability to degrade polylactic acid (PLA), which is a biodegradable aliphatic polyester, in soil [39]. It was also abundant in rhizosphere soil microbial communities in a Mediterranean mountain ecosystem [40]. 

**Figure 6:**
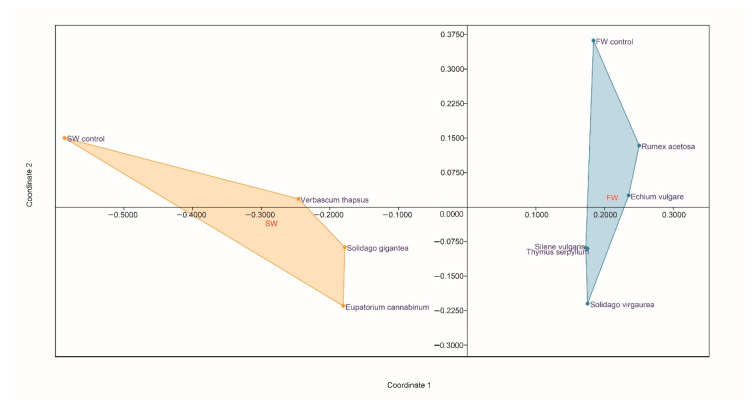
Non-metric multidimensional scaling (NMDS) for the microbial community structure in FW and SW soils.

Non-metric multidimensional scaling (NMDS) analyses revealed substantial genetic differences between FW and SW samples, but also confirmed strong spontaneous plant-driven shift in the structure of bacterial communities, apparently assisting processes of plant adaptation to the harsh conditions (Figure 6). In the FW samples, relatively similar community structures were observed for *T. serpyllum*, *S. vulgaris*, and to some extent *S. virgaurea*, whereas *E. vulgare* and *R. acetosa* comprised another relatively consistent group of plant species. It is possible that specific structure of microbial populations in rhizosphere of these two plants contributed to their resistance to high uptake of PTTE. The bulk soil of SW was genetically different to all the root zone soils, among which *V. thapsus* and *S. gigantea* exhibited more inter-similarity than the *E. cannabinum*.

## 3. Materials and Methods

### 3.1. Description of the Experimental Site

The two experimental sites involved the long-term deposits of two types of Zn-Pb smelter waste. The site located in Dolki village, near Piekary Slaskie, Poland (50°21′19″ N; 19°00′17″ E), is a deposit of flotation waste (FW) formed in 1915–1930 through the deposition of flotation residues from the mining and processing of Zn-Pb ores, mainly fine metal rich dolomite [5]. It is almost barren at the top and the slopes are partly covered with spontaneous vegetation. It is subject to water and wind erosion processes during intensive rainfall or long dry periods, respectively, affecting nearby arable land parcels (Figure S2). On three sides, the heaps are surrounded by arable fields that are cropped with wheat, cabbage, sugar beet, corn, or potato, depending on the year. The nearest residential buildings are located less than 100 m from the pile, and the centre of Dolki village is approximately 250 m from its edge. Previous studies have shown that metal content in the waste was extremely high—a mean 9.3% Zn total content with maximum value 17.2%, average Pb content of 1.75%, and Cd 386 mg kg^−1^. Waste pH was slightly alkaline (7.3–7.7 in water) [5]. 

The second location was the slag waste pile from a decommissioned ore smelting plant located in Piekary Slaskie, Poland (50°21′88″ N; 18°58′19″ E). The deposit mainly contains the slag waste (SW) from the Waeltz smelting process, which took place in long, spinning furnaces used to enrich low Zn and Pb ores [3]. The waste is still very high in Zn, Pb, and Cd—the average content is 3.82%, 1.07%, and 361 mg kg^−1^, respectively [5]. Waste pH is generally neutral. The site is barren of vegetation, with very rare spontaneous plant species growing in single locations. It therefore constitutes a substantial environmental and health hazard through wind erosion and metal leaching into ground and surface waters. The site is located approximately 350 m from the nearest residential buildings, approximately 350 m from hobby gardens, and approximately 350 m from the nearest arable land. 

The climate in Piekary Slaskie is cold and temperate. The rainfall is relatively high, with an annual average of 673 mm. The driest month is February, with precipitation of 32 mm. Most precipitation falls in July, which is on average 94 mm. According to the Köppen-Geiger climate classification, the climate is Dfb. The average temperature in Piekary Slaskie is 8.3 °C. Winds blow mostly from the west and northwest. The average monthly temperatures in the winter (January) and summer (July) periods, as calculated based on long term data, are 1.5 °C and 18 °C, respectively [41].

### 3.2. Waste Pile Top Layer and Plant Sampling

**Figure 7:**
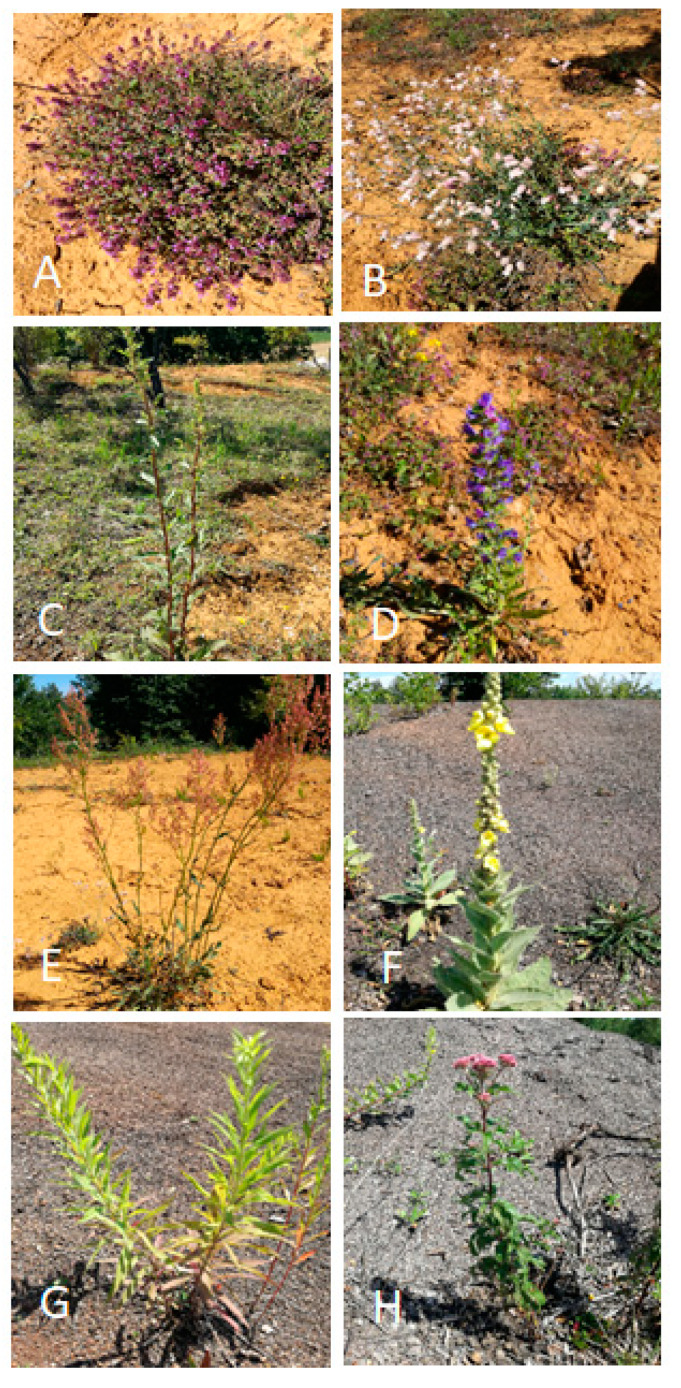
Plant species growing on FW and SW sites, selected for soil and plant sampling: Flotation waste—(**A**) *T. serpyllum*, (**B**) *S. vulgaris*, (**C**) *S. virgaurea*, (**D**) *E. vulgare*, (**E**) *R. acetosa*, Slag waste—(**F**) *V. thapsus*, (**G**) *S. gigantea*, (**H**) *E. cannabinum.*

Soil samples were collected in June 2018 from the root zone of eight different plant species spontaneously growing on the two waste deposits. Species were selected because they were the most abundant in the period of sampling and performing relatively well. The soil of the root zone of the following plant species was sampled: flotation waste (FW) (5 species)—*Thymus serpyllum*, *Silene vulgaris*, *Solidago virgaurea*, *Echium vulgare*, and *Rumex acetosa*; and slag waste (SW) (3 species)—*Verbascum thapsus*; *Solidago gigantea*, *Eupatorium cannabinum* (Figure 7). Soil samples were collected from root zone of 3 individual plants of each plant species, growing in different sectors of a waste heap. These 3 samples constituted 3 replicates of root zone of a given plant species.

Soil samples were collected from the root zone of individuals growing away from other plant species to avoid the influence of other plant species on soil microorganisms. The depth of sampling was adjusted to the root depth, but did not exceed 15 cm. Control samples were taken from both FW and SW waste from barren areas in order to collect soils unaffected by plants. The total number of samples was 30 (8 plant species × 3 replicates plus 2 control soils (FW and SW) × 3 replicates).

An approximate 1.5 kg sample was taken from the root system of each plant and thoroughly mixed, homogenised, and transported to the laboratory, where the material was sieved through a 2 mm mesh and subdivided into two portions: one to be dried for chemical analysis and the second stored fresh in the refrigerator at ±4 °C for microbiological and biochemical analysis. Control samples were collected from both waste pile sites from the areas without any vegetation.

Samples of aboveground parts of individual plant species were collected at different developmental stages: *S. virgaurea* and *S. gigantea* in the flower bud phase, *V. thapsus* and *E. cannabinum* at the beginning of flowering, *T. serpyllum*, *S. vulgaris,* and *E. vulgare* at full flowering phase, and *R. acetosa* after flowering. The plant samples were collected for analysis of plant chemical composition. The plant material was washed in deionised water, dried, ground, and subjected to the determination of trace element and macro-element contents.

### 3.3. Microbiological Soil Analysis

The activities of three enzymes (dehydrogenases, acidic, and alkaline phosphatases) were measured using standard protocols in order to characterise the biochemical activity of the waste heaps [42]. The determination of dehydrogenases was performed according to Casida et al. [43] using the colorimetric method, with TTC (triphenyltetrazolium chloride) as a substrate, after 24 h of incubation at 37 °C. Alkaline and acid phosphatase activities were measured by the colorimetric method using PNP (sodium p-nitrophenylphosphate) after 1 h of incubation at 37 °C at 410 nm wavelength [44]. The total count of cultivable bacteria [45], ammonification bacteria [46], Azotobacter spp. [47], and the count of cultivable fungi [48] was determined using the plate dilution method. The plates were incubated at 28 °C and the number of colonies was counted after 3–5 days of growth. Recovery Replicates for Laboratory Control Samples method was used for uncertainty calculation for the count methods. Expanded uncertainty (EU) values were as follows: 10, 8, 12, 12, and 8% for bacteria, fungi, oligotrophic bacteria, copiotrophic bacteria, ammonification bacteria, respectively. For all collected samples, biochemical and microbiological measurements were done in three technical replicates.

The metabolic profile (phenotypic) assessment of soil samples was carried out using the BIOLOG EcoPlate^®^ System (Biolog TM, Hayward, CA, USA). The method is based on the direct inoculation of BIOLOG plates, containing a range of carbon sources with soil suspension. One gram of soil was transferred to sterile 0.9% NaCl, shaken for 30 min, and then cooled for 30 min to 4 °C. Subsequently, 120 mm3 of the suspension was transferred to each well of the EcoPlate and incubated at 25 °C. A change in the purple tetrazolium colour is an indicator of the degradation of the given carbon source. Absorbance at 590 nm was measured using BIOLOG Microstation after various periods of incubation.

For soil genetic diversity, an analysis of 10 composite samples, representing each of 8 plant species and controls, were prepared. Parts of homogenous replicates (approx. 50 g) were pooled together and subjected to the DNA extraction. Total DNA was extracted from the soil sample using the FastDNA™ SPIN Kit for soil (MPBiomedical), and the V3–V4 region of the 16S rRNA gene was sequenced using 341F and 785R primers [49] at Genomed S.A. (Warsaw, Poland), in 2 bp × 250 bp paired-end technology using the Illumina MiSeq system. Demultiplexed fastq files were processed using the DADA2 (1.12) package [50] in R software (3.6.0) [51]. Forward and reverse reads were trimmed to 250 bp, and primer sequences were removed from all reads. The filtering parameters were as follows: maxN = 0, maxEE for both reads = 2, truncQ = 2. MaxEE corresponds to the maximum expected errors. Expected errors are calculated from the quality score (EE = sum (10^(−Q/10)). TruncQ = 2 parameter truncate reads at the first instance of a quality score less than or equal to two. MaxN is the maximum number of accepted “N” bases. Error rates were estimated by learnErrors using one billion reads. Sequences were de-replicated using derepFastq with default parameters, and the exact sequence variants were resolved using dada. RemoveBimeraDenovo was then used to remove chimeric sequences. After the filtration steps, 69% (mean = 137,758) of the reads were left for further analysis. Taxonomy was assigned against the latest version of the GTDB database [52,53,54], released 17 July 2020, using IDTAXA [54] on the sequences table resulting from the DADA2 workflow described above. The results were converted and imported into the phyloseq (1.22.3) package [55]. Sequences belonging to the chloroplast or mitochondrial DNA were removed. Subsequently, for further analysis, the total number of reads for the individual taxa was converted to a percentage, assuming the sum of all taxa in the individual samples as 100%.

For genus assignment, the amplicon sequence variant (ASV) approach was utilized. On average, 19.86% of all reads which were classified to the genus level were further aggregated, and their abundances summed. The operating taxonomic units OTUs were generated and used for unclassified reads in order to decrease the number of total taxa members. Unclassified reads were clustered using vsearch [56] implemented in seed software, version 2.1 [57] at a 97% similarity level. Each of the 5672 clustered groups of unclassified reads were then uniquely named and merged with the previous table (containing reads classified to the genus level). This approach enabled the statistical processing of the true alpha and beta diversity, regardless of whether there was a sequence in the reference database or not. In total, 6060 unique taxa (at the genus level plus unclassified cluster) were detected in all samples.

### 3.4. Chemical Soil Analysis and Plant Analysis

The air-dried soil samples were ground in a porcelain mortar and sieved through a 2 mm diameter screen, then milled, homogenised, and stored in paper bags at 20 ± 1 °C until the analysis was performed. Certified reference materials (CRM) and internal laboratory standards were analysed for the control of chemical determinations.

Air dried samples were subjected to pH using a combined glass electrode in a slurry with a 1:2 *v*/*v* soil/water ratio. Electrical conductivity was measured in filtrates of a 1:5 soil/water slurry at 25 °C. Organic matter (OM) content was measured by loss on ignition in a muffle oven at 480 °C within 16 h. Total trace element contents were measured after digestion of a sample in a 3:1 mixture of concentrated HNO_3_:HCl in Teflon PFA vessels in a microwave accelerated reaction system (MarsXpress; CEM Corp., Matthews, NC, USA) followed by measurements of elements in extracts by ICP-MS (Agilent 7500ce). 

Mineral nitrogen (N) extractability was measured by flow spectrometry after extraction with 1 M K_2_SO_4_ using QuAAtro39 analyser (Seal Analytical, Norderstedt, Germany). Available phosphorus (P) was measured using the Egner-Riehm colorimetric method using extraction with calcium lactate (0.02 M) in diluted HCl (0.01 M), followed by colorimetric measurement in the Perkin Elmer Lambda 45 Spectrometer, based on the reaction with ammonium molybdate. Available potassium (K) was measured after the same extraction by AAS, using AAnalyst 800 (Perkin Elmer, Waltham, MA, USA).

Metals solubility was analysed by extraction in 0.01 M calcium chloride (1:10 solution/water ratio, shaken for 2 h at room temperature) and subsequent measurement of metal concentrations by ICP-MS. 

Ground plant tissues were digested in concentrated HNO_3_ in Teflon PFA vessels in a microwave accelerated reaction system (MarsXpress; CEM Corp., Matthews, NC, USA) followed by measurements of elements in extracts by ICP-MS (Agilent 7500ce). The bioconcentration factor (BF) was calculated for PTTE as a ratio of metal content in aboveground plant tissues to total element content in soil.

### 3.5. Statistical Analysis

In order to compare the plant effects on the soil chemical and microbiological properties of root zone soil, the results were expressed as means and the differences were considered significant at *p* < 0.05. When the data was normally distributed and the variance was homogeneous, as based on the Shapiro–Wilk and the Levene tests, respectively, the one-way analysis of variance (ANOVA) was applied to entire dataset, combining both wasteland sites. Differences between the plant species were identified using the Tukey test.

Pearson’s correlation coefficients were calculated to evaluate significance of relationships between variables. Multiple linear regression models were generated to find soil chemical parameters (independent variables) that most influenced abundance of culturable microorganisms and activity of soil enzymes (dependent variables) in root zone of spontaneous plants. The ordinary least squares (OLS) regression analysis using stepwise backward approach was applied for estimation of model parameters. Control soils were not taken to the analysis in order to: (i) avoid strong impact of low activity in control samples on the models; (ii) learn how much variability in dependent variables in root zone is connected to soil chemical characteristics. Graphical analysis of the residual normality plot was done for all models to test whether the model residuals meet the basic assumptions of the OLS method. All these analyses were carried out using Statistica v.13.0 (TIBCO Software Inc., Palo Alto, CA, USA).

The Biolog Ecoplate-derived functional diversity indices of microorganisms activity, such as AWCD, the Shannon diversity index (H), Evenness index (J), and Richness index (R) were calculated for data after 144 h. Variation in the relationship between plant species and carbon source utilization and extractable PTTE was explored using principal component analysis (PCA). PCA is a mathematical protocol converting a set of observations of possibly correlated variables into linearly uncorrelated variables called principal components. PCA is a common technique used to describe patterns of variation within multi-dimensional datasets [58]. Before analysis, all data was centred and log transformed. PCA analyses and H′, J’ and R calculations were performed using the MVSP- Multivariate Statistical Package [59].

The data obtained in the next-generation sequencing (NGS) procedure was subjected to non-metric multidimensional scaling (NMDS) in Past 3.25 software [60]. The NMDS procedure revealed the strass value 0.07.

## 4. Conclusions

Our study puts a new light on the complexity of survival and adaptation mechanisms of pioneer plant species colonizing metalliferous waste, which initiates the soil formation process. It is obvious from the data obtained that appearance of spontaneous plants on the waste piles stimulates shifts in soil chemical and biological properties through plant-microbial interactions. It is apparent that these interactions lead to mobilization of nutrients such as P and K—extractability of these nutrients in spots colonized by studied plant species was much higher as compared to barren control. Similar interactions are responsible for much greater accumulation of nitrogen in the colonized spots. This phenomenon is among critical factors for the survival of vegetation in these chemically stressed environments. Species also vary in their ability to impact the accumulation of nitrogen in the root zone—*S. virgaurea* and *T. serpyllum* supports highest production of N-NH_4_ among all studied plants.

It is worth noting that regardless of differences between species in C utilization patterns, there is a relatively wide range of C sources each plant specimen can utilize in interaction with microorganism—this also contributes to survival mechanisms, explaining why these particular plants are first to colonize metalliferous toxic materials.

Our study provides new data on the microbial and functional characteristics of soil under different plant species, spontaneously grown on toxic metal waste deposits. Regardless of initial heterogeneity of the physical and chemical properties of the two studied waste materials, it is evident that over the years from site decommissioning, spontaneous vegetation led to the formation of soil-like material. This industrial soil exhibits habitat functions regardless of its initial and current chemical stress for plants. It is apparent that the initial properties of the two waste types, which cannot be assessed today, played a key role in controlling the composition of spontaneous vegetation.

Our comparative analysis of chemical and microbial characteristics of root zone soil under different plant species and these of barren waste indicate that colonization of metalliferous waste piles is driven by the adaptability of the individual specimen to the chemical and physical stress. On the other hand, plant species and microbial communities of their root zone have also contributed to the transformation of toxicity indicators and other soil biological and chemical parameters. These complex interactions between plants, microbiome, and substrate occurring over the years produced a current set of unique characteristics of the root zone soil which is specific to an individual plant specimen.

## Figures and Tables

**Table 1:** Average chemical characteristics of the flotation waste (FW) and slag waste (SW) soils.

Plant Species	pH	Organic Matter (OM)	Available P (P_2_O_5_)	Available K (K_2_O)	N-NH_4_
		%	mg kg^−1^		
*T. serpyllum*	8.00 ± 0.08 ^1,a2^	14.0 ± 0.3 ^ab^	17.8 ± 0.3 ^a^	176 ± 2.9 ^bc^	8.20 ±0.7 ^c^
*S. vulgaris*	8.13 ± 0.05 ^ab^	13.0 ±1.0 ^a^	19.6 ± 0.1 ^ab^	194 ± 5.7 ^c^	7.07 ± 2.1 ^bc^
*S. virgaurea*	8.13 ± 0.05 ^ab^	12.7 ±2.3 ^a^	18.5 ± 0.1 ^a^	172 ± 1.6 ^abc^	9.88 ± 0.6 ^c^
*E. vulgare*	8.23 ± 0.05 ^abc^	12.1 ± 0.3 ^a^	18.0 ± 0.1 ^a^	67 ± 2.1 ^ab^	3.44 ± 1.4 ^a^
*R. acetosa*	8.17 ± 0.05 ^ab^	12.4 ± 0.3 ^a^	15.1 ± 0.3 ^a^	50 ± 1.0 ^a^	4.02 ± 1.5 ^ab^
FW control	8.23 ± 0.05 ^abc^	11.8 ± 0.7 ^a^	10.1 ± 0.1 ^a^	67 ± 1.0 ^ab^	2.01 ± 0.3 ^a^
*V. thapsus*	8.23 ± 0.05 ^abc^	18.1 ± 0.7 ^b^	31.8 ± 0.6 ^b^	160 ± 6.6 ^abc^	2.61 ± 1.6 ^a^
*S. gigantea*	8.27 ± 0.09 ^abc^	11.9 ± 3.7 ^a^	18.0 ± 0.9 ^a^	206 ± 4.4 ^c^	2.27 ± 0.3 ^a^
*E. cannabin.*	8.43 ± 0.09 ^bc^	16.0 ± 1.1 ^ab^	22.3 ± 0.3 ^ab^	111 ± 6.6 ^abc^	3.86 ± 0.6 ^ab^
SW control	8.53 ± 0.05 ^c^	16.7 ± 1.5 ^ab^	9.8 ± 0.5 ^a^	91 ± 1.0 ^abc^	1.91 ± 0.1 ^a^

^1^ Standard Deviation. ^2^ Means marked with the same letter did not differ significantly across the plant species (*p* < 0.05, *n* = 3) according to the Tukey test.

**Table 2:** Total metal contents in FW and SW soils (mg kg^−1^).

Plant Species	Cu	Zn	As	Cd	Pb	Mg	K	Ca	Fe
*T. serpyllum*	51 ± 22 ^1, a2^	85,501 ± 2950 ^c^	2091 ± 300 ^bcd^	418 ± 8 ^ab^	18,552 ± 1430 ^ab^	19,722 ± 2160 ^a^	3077 ± 198 ^bc^	39,805 ± 4990 ^a^	255,884 ± 6420 ^ab^
*S. vulgaris*	35 ± 6 ^a^	108,838 ± 5160 ^e^	2517 ± 147 ^d^	538 ± 25 ^b^	23,327 ± 1189 ^ab^	23,959 ± 3600 ^ab^	3726 ± 317 ^cd^	47,102 ± 7670 ^a^	288,467 ± 9700 ^b^
*S. virgaurea*	49 ± 13 ^a^	93,037 ± 4700 ^cd^	2293 ± 146 ^cd^	468 ± 17 ^ab^	20,824 ± 846 ^ab^	20,201 ± 1108 ^a^	4291 ± 254 ^d^	41,031 ± 410 ^a^	273,304 ± 14,560 ^ab^
*E. vulgare*	34 ± 3 ^a^	105,111 ± 2440 ^de^	2562 ± 186 ^d^	495 ± 21 ^ab^	22,528 ± 1164 ^ab^	23,603 ± 2510 ^ab^	4081 ± 516 ^cd^	45,033 ± 5130 ^a^	292,675 ± 5530 ^b^
*R. acetosa*	30 ± 5 ^a^	112,211 ± 4330 ^e^	2505 ± 75 ^d^	556 ± 20 ^b^	24,130 ^b^ ± 290	26,992 ± 2630 ^abc^	3811 ± 406 ^cd^	52,559 ± 5470 ^a^	325,362 ± 16,800 ^b^
FW control	24 ± 2 ^a^	101,716 ± 2260 ^de^	2351 ± 364 ^cd^	514 ± 20 ^b^	21,286 ± 583 ^ab^	25,333 ± 1850 ^ab^	2378 ± 258 ^ab^	51,918 ± 5530 ^a^	297,958 ± 5560 ^b^
*V. thapsus*	2170 ± 360 ^b^	18,292 ± 2770 ^a^	1835 ^d^ ± 230 ^abc^	233 ± 127 ^a^	11,515 ± 2690 ^a^	31,732 ± 2790 ^abc^	2389 ± 373 ^ab^	124,118 ± 14,600 ^b^	254,603 ± 21,010 ^ab^
*S. gigantea*	2162 ± 260 ^b^	18,542 ± 3700 ^a^	1454 ± 293 ^ab^	311 ± 121 ^ab^	12,392 ± 3440 ^ab^	35,691 ± 4510 ^bc^	2108 ± 338 ^ab^	152,248 ± 10,900 ^b^	245,532 ± 29,680 ^ab^
*E. cannabinum*	1829 ± 137 ^b^	25,990 ± 1130 ^ab^	1284 ± 101 ^a^	331 ± 107 ^ab^	11,956 ^a^ ± 2110 ^b^	38,577 ± 4770 ^c^	1794 ± 153 ^a^	128,312 ± 15,400 ^b^	194,861 ± 13,920 ^a^
SW control	1931 ± 159 ^b^	32,564 ± 6600 ^b^	1564 ± 238 ^abc^	593 ± 236 ^b^	20,894 ^a^ ± 3726 ^b^	29,627 ± 2760 ^abc^	2237 ± 130 ^ab^	126,584 ± 14,560 ^b^	195,606 ± 12,790 ^a^

^1^ Standard Deviation. ^2^ Means marked with the same letter did not differ significantly across the plant species (*p* < 0.05, *n* = 3) according to the Tukey test.

**Table 3:** Chemical composition of plants inhabiting FW and SW soils (mg kg^−1^) and bioconcentration factors (BF) for potentially toxic trace elements (PTTEs).

Plant Species	Zn		Cd		Pb		As		Fe	Ca
	Content	BF	Content	BF	Content	BF	Content	BF	Content	Content
*T. serpyllum*	6337 ^b1^	0.07 ^b^	52.7 ^b^	0.13 ^b^	1626 ^b^	0.09 ^b^	134 ^b^	0.06 ^b^	12,231 ^b^	13,833 ^a^
*S. vulgaris*	2847 ^ab^	0.03 ^bc^	15.2 ^a^	0.03 ^d^	506 ^a^	0.02 ^d^	56 ^ab^	0.02 ^c^	5528 ^ab^	9009 ^a^
*S. virgaurea*	800 ^a^	0.01 ^c^	5.0 ^a^	0.01 ^e^	161 ^a^	0.01 ^d^	15 ^a^	0.01 ^c^	1488 ^a^	9471 ^a^
*E. vulgare*	20,870 ^c^	0.20 ^a^	113 ^c^	0.23 ^a^	4351 ^c^	0.19 ^a^	473 ^c^	0.18 ^a^	43,859 ^c^	40,276 ^b^
*R. acetosa*	5103 ^ab^	0.05 ^b^	22.4 ^a^	0.04 ^d^	860 ^ab^	0.04 ^c^	96 ^ab^	0.04 ^bc^	9160 ^ab^	18,484 ^a^
*V. thapsus*	954 ^a^	0.05 ^b^	10.4 ^a^	0.04 ^d^	470 ^a^	0.04 ^c^	41 ^ab^	0.02 ^c^	3889 ^ab^	10,252 ^a^
*S. gigantea*	236 ^a^	0.01 ^c^	4.6 ^a^	0.01 ^e^	157 ^a^	0.01 ^d^	9 ^a^	0.01 ^c^	632 ^a^	8381 ^a^
*E. cannabinum*	417 ^a^	0.02 ^c^	26.6 ^ab^	0.08 ^c^	171 ^a^	0.01 ^d^	15 ^a^	0.01 ^c^	1419 ^a^	13,003 ^a^

^1^ Means marked with the same letter did not differ significantly across the plant species (*p* < 0.05, *n* = 3) according to the Tukey test.

**Table 4:** Enzyme activities in FW and SW soils.

Plant Species	Dehydrogenases	Acidic Phosphatase	Alkaline Phosphatase
	µg TPF g^−1^ DM 24 h^−1^	µg PNP g^−1^ DM h^−1^	µg PNP g^−1^ DM h^−1^
*T. serpyllum*	24.4 ^bc^	32.6 ^c^	73.2 ^b^
*S. vulgaris*	13.6 ^abc^	23.1 ^abc^	40.9 ^ab^
*S. virgaurea*	18.7 ^bc^	30.9 ^bc^	71.9 ^b^
*E. vulgare*	2.9 ^a^	19.8 ^abc^	25.2 ^a^
*R. acetosa*	3.7 ^ab^	20.0 ^abc^	28.2 ^a^
FW control	0.1 ^a^	15.6 ^abc^	11.5 ^a^
*V. thapsus*	0.5 ^a^	9.0 ^a^	17.1 ^a^
*S. gigantea*	5.2 ^ab^	14.8 ^ab^	24.2 ^a^
*E. cannabinum*	1.0 ^a^	10.6 ^a^	23.2 ^a^
SW control	0.1 ^a^	12.8 ^a^	11.4 ^a^

Means marked with the same letter did not differ significantly across the plant species (*p* < 0.05, *n* = 3) according to the Tukey test.

**Table 5:** Count of cultivable microorganisms in FW and SW soils.

Plant Species	Total Bacteria	Total Fungi	Oligotrophic Bacteria	Copiotrophic Bacteria	Ammonification Bacteria
	10^8^	10^4^	10^7^	10^7^	10^7^
*T. serpyllum*	8.1 ^ab^	22.4 ^cd^	45.8 ^b^	24.9 ^ab^	0.3 ^a^
*S. vulgaris*	11.7 ^b^	45.2 ^d^	124 ^c^	54.0 ^b^	0.4 ^a^
*S. virgaurea*	4.1 ^a^	22.1 ^cd^	31.1 ^b^	21.2 ^ab^	0.3 ^a^
*E. vulgare*	7.1 ^a^	14.6 ^bc^	37.1 ^b^	20.8 ^ab^	0.4 ^a^
*R. acetosa*	11.6 ^b^	15.1 ^bc^	50.6 ^b^	46.9 ^b^	57.9 ^b^
FW control	2.1 ^a^	0.4 ^a^	6.4 ^a^	4.0 ^a^	8.5 ^a^
*V. thapsus*	15.5 ^b^	9.9 ^b^	44.6 ^b^	34.5 ^ab^	41.8 ^b^
*S. gigantean*	37.2 ^c^	7.7 ^b^	221 ^d^	111 ^c^	184 ^c^
*E. cannabinum*	13.4 ^ab^	13.7 ^bc^	34.6 ^b^	22.6 ^ab^	35.2 ^b^
SW control	3.6 ^a^	3.0 ^ab^	13.8 ^a^	10.5 ^a^	18.4 ^ab^

Means marked with the same letter did not differ significantly across the plant species (*p* < 0.05, *n* = 3) according to the Tukey test.

**Table 6:** Stepwise regression model fitting results for microbial abundance and enzyme activities in FW and SW soils (*n* = 24).

Microbial Parameter	Independent Variable	Coefficient	Significance Level	Model R^2^
Bacteria	Total Cu Total Fe Extr. As Constant	0.022 0.000 −153.5 −39.8	0.011	0.33
Fungi	Avail. K Total Mg Total Ca Constant	2.14 0.003 −0.0006 −41.2	<0.001	0.52
Oligotrophs	Avail. K Total Cu Total K Total Ca Total Fe Extr. As Constant	7.93 0.29 −0.07 −0.00 0.00 −2454 −266	<0.001	0.84
Copiotrophs	Avail. K Total Cu Total Fe Extr. As Constant	3.3 0.089 0.001 −896 −191.7	<0.001	0.61
Ammonification bacteria	Avail. P Total Cu Total Ca Total Fe Extr. As Constant	−51.23 0.34 −0.00 0.00 −1947 −116.9	<0.001	0.85
Acidic phosphatase	OM N-NH_4_ Total Mg Constant	−0.55 1.63 −0.0005 34.05	<0.001	0.89
Alkaline phosphatase	N-NH_4_ Total Mg Total Ca Constant	6.74 −0.002 0.0003 37.07	<0.001	0.88
Dehydrogenase	EC Total Cu Total As Extr. Cd Constant	0.029 −0.004 −0.014 11.08 22.37	<0.001	0.86

**Table 7:** Biodiversity indices of FW and SW soils based on substrate utilisation pattern in the Biolog EcoPlate at time 144 h.

Plant Species	Biodiversity Indices			
	AWCD	Shannon Diversity Index (H’)	Evenness Index (J’)	Richness (R)
*T. serpyllum*	1.12 ^d^	3.24 ^a^	0.95 ^ab^	30.7 ^a^
*S. vulgaris*	1.29 ^cd^	3.27 ^a^	0.95 ^ab^	30.7 ^a^
*S. virgaurea*	1.20 ^cd^	3.26 ^a^	0.97 ^b^	29.0 ^a^
*E. vulgare*	1.57 ^abc^	3.23 ^a^	0.96 ^ab^	29.3 ^a^
*R. acetosa*	1.12 ^d^	3.26 ^a^	0.96 ^ab^	30.0 ^a^
FW control	0.16 ^e^	2.46 ^b^	0.82 ^c^	20.3 ^b^
*V. thapsus*	1.98 ^a^	3.33 ^a^	0.97 ^b^	30.7 ^a^
*S. gigantea*	1.47 ^bcd^	3.25 ^a^	0.95 ^ab^	31.0 ^a^
*E. cannabinum*	1.76 ^ab^	3.39 ^a^	0.99 ^a^	30.7 ^a^
SW control	0.32 ^e^	2.90 ^a^	0.87 ^bc^	28.0 ^a^

Means marked with the same letter did not differ significantly across the plant species (*p* < 0.05, *n* = 3) according to the Tukey test.

## Supplementary Materials

The following are available online. Table S1: CaCl_2_-extractable metal contents in FW and SW soils, Table S2: Correlation coefficients for relationships between soil chemical variables and microbial abundance and activity, Table S3: Results of PCA based on contents of extractable trace elements and intensity of C substrate utilization, Table S4: Correlation coefficients for relationships between metabolic activity parameters and extractable forms of trace elements, Figure S1: Heat maps of the metabolic profile of microorganisms based on utilisation of the 31 C sources using the EcoPlate method after 144 h incubation, Figure S2: The border between the smelter waste pile (Dolki) and the arable land (cabbage field)—year 2018.
