# Supplementary material for: Activity and Diversity of Microorganisms in Root Zone of Plant Species Spontaneously Inhabiting Smelter Waste Piles
Source: Molecules. 2020 Nov 30;25(23):5638. doi: 10.3390/molecules25235638 (PMC7730207; doi:10.3390/molecules25235638)
Supplement: Supplementary file 1 [file molecules-25-05638-s001.pdf]

Table S1. $CaCl_2$-extractable trace element contents in FW and SW soils (mg kg^{-1}).

Plant species	Cu	Zn	As	Cd	Pb
T. serpyllum	0.01^{a1} (0.001)[2]	37.6^{b} (5.3)	0.03^{a} (0.004)	2.10^{b} (0.4)	1.59^{c} (0.4)
S. vulgaris	0.01^{a} (0.001)	36.5^{b} (3.8)	0.03^{a} (0.005)	2.16^{b} (1.0)	1.59^{c} (0.1)
S. virgaurea	0.01^{a} (0.004)	29.9^{b} (4.8)	0.03^{a} (0.003)	2.24^{b} (0.08)	1.30^{c} (0.2)
E. vulgare	0.01^{a} (0.001)	32.3^{b} (2.1)	0.02^{a} (0.001)	1.34^{ab} (0.4)	1.35^{c} (0.1)
R. acetosa	0.01^{a} (0.001)	34.2^{b} (2.8)	0.02^{a} (0.002)	1.26^{ab} (0.2)	1.23^{c} (0.2)
FW control	0.01^{a} (0.001)	27.2^{b} (1.9)	0.02^{a} (0.006)	0.82^{ab} (0.1)	0.99^{bc} (0.2)
V. thapsus	0.07^{bc} (0.01)	1.05^{a} (0.2)	0.22^{b} (0.09)	0.11^{a} (0.03)	0.19^{ab} (0.06)
S. gigantea	0.09^{c} (0.02)	0.86^{a} (0.6)	0.14^{ab} (0.04)	0.14^{a} (0.1)	0.17^{a} (0.1)
E. cannabinum	0.10^{c} (0.04)	1.60^{a} (0.5)	0.13^{ab} (0.03)	0.28^{a} (0.1)	0.22^{ab} (0.1)
SW control	0.02^{ab} (0.01)	1.81^{a} (0.1)	0.09^{a} (0.006)	0.33^{a} (0.1)	0.32^{ab} (0.1)

[1]Means marked with the same letter did not differ significantly across the plant species ($p < 0.05$, $n = 3$) according to the Tukey test

[2]Standard Deviation in parenthesis

Table S2. Correlation coefficients for relationships between soil chemical variables and microbial abundance and activity (n=24).

Soil variable	Bacteria	Fungi	Oligotrophs	Copiotrophs	Amonification bacteria	Dehydrogenase	Acidic phosphatase	Alkaline phosphatase
pH	0,09	-0,26	-0,25	-0,18	-0,09	*-0,67*	*-0,76*	*-0,59*
EC	0,32	-0,18	*0,72*[1]	*0,63*	*0,77*	-0,05	-0,17	-0,23
OM	0,17	0,01	-0,32	-0,2	-0,22	-0,18	-0,34	-0,12
NH4	-0,38	0,40	-0,17	-0,26	-0,38	*0,86*	*0,84*	*0,91*
NO3	0,17	0,31	-0,16	-0,14	-0,22	0,02	-0,05	0,16
Av[2] P	0,22	-0,15	-0,24	-0,17	-0,18	-0,33	*-0,50*	-0,29
Av K	0,30	0,40	*0,45*	0,37	0,25	*0,41*	0,24	0,35
Cu t[3]	*0,41*	*-0,41*	0,18	0,23	*0,41*	*-0,50*	*-0,75*	*-0,54*
Zn t	*-0,44*	*0,41*	-0,19	-0,23	*-0,43*	0,36	*0,64*	0,39
As t	-0,28	0,35	-0,06	-0,09	-0,28	0,32	*0,57*	0,33
Pb t	-0,40	*0,41*	-0,27	-0,27	*-0,47*	0,24	*0,54*	0,31
Mg t	0,25	-0,36	0,01	0,03	0,19	*-0,69*	*-0,85*	*-0,72*
K t	-0,35	0,20	-0,29	-0,31	*-0,45*	0,26	*0,55*	0,33
Ca t	*0,45*	*-0,41*	0,19	0,23	*0,42*	*-0,56*	*-0,78*	*-0,60*
Fe t	0,02	0,16	0,09	0,08	-0,02	0,09	0,34	0,06
Cu c[4]	0,39	-0,29	0,16	0,18	0,37	*-0,42*	*-0,60*	-0,40
Zn c	*-0,41*	*0,42*	-0,17	-0,21	*-0,42*	*0,55*	*0,78*	*0,56*
As c	0,24	-0,34	-0,08	-0,05	0,1	*-0,47*	*-0,68*	*-0,47*
Cd c	-0,38	0,36	-0,19	-0,23	-0,38	*0,80*	*0,86*	*0,82*
Pb c	-0,40	*0,42*	-0,19	-0,27	*-0,47*	*0,52*	*0,74*	*0,52*

[1] numbers in italic are attributed to statistically significant relationships at $p<0.05$

[2] av: available

[3] t: total

[4] c: Ca-chloride extractable

Table S3. Results of PCA based on contents of extractable trace elements and intensity of C substrate utilisation

Variables	Axis 1	Axis 2
Eigenvalues and variance (%) explained by the first two PCA axes		
Eigenvalues	6.72	3.69
Percentage	61.06	33.63
Cumulative percentage	61.06	94.69
Loading components for each variable associated with the two axes		
Extractable Cu	-0.314	-0.253
Extractable Zn	0.238	0.397
Extractable As	-0.295	-0.299
Extractable Cd	0.186	0.441
Extractable Pb	0.220	0.424
Amines and Amides	-0.338	0.093
Aminoacids	-0.320	0.278
Carboxylic and acetic acids	-0.341	0.235
Carbohydrates	-0.313	0.293
Polimers	-0.353	0.184
AWCD	-0.345	0.232

Table S4. Correlation coefficients for **r**elationships between metabolic activity parameters and extractable forms of trace elements

	Cu	Zn	As	Cd	Pb
Amines and Amides	*0.71*[1]	-0.42	0.56	-0.36	-0.42
Aminoacids	0.48	-0.14	0.41	-0.05	-0.12
Carboxylic and acetic acids	*0.66*	-0.31	*0.63*	-0.21	-0.29
Carbohydrates	0.45	-0.07	0.39	0.06	-0.03
Polimers	*0.64*	-0.35	*0.65*	-0.20	-0.31
AWCD	0.57	-0.21	0.52	-0.09	-0.18

[1] numbers in italic are attributed to statistically significant relationships at $p<0.05$

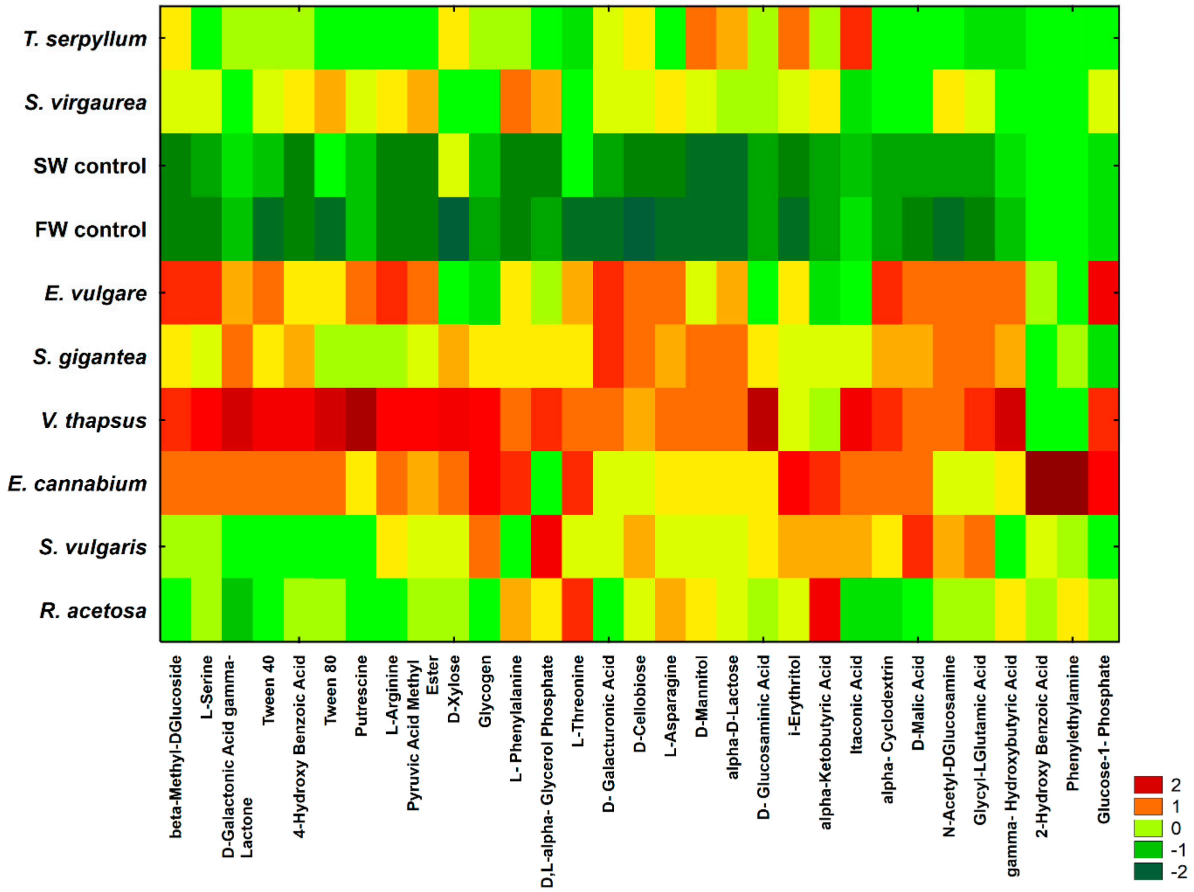

Figure S1. Heat maps of the metabolic profile of microorganisms based on utilisation of the 31 C sources using the EcoPlate method after 144 h incubation

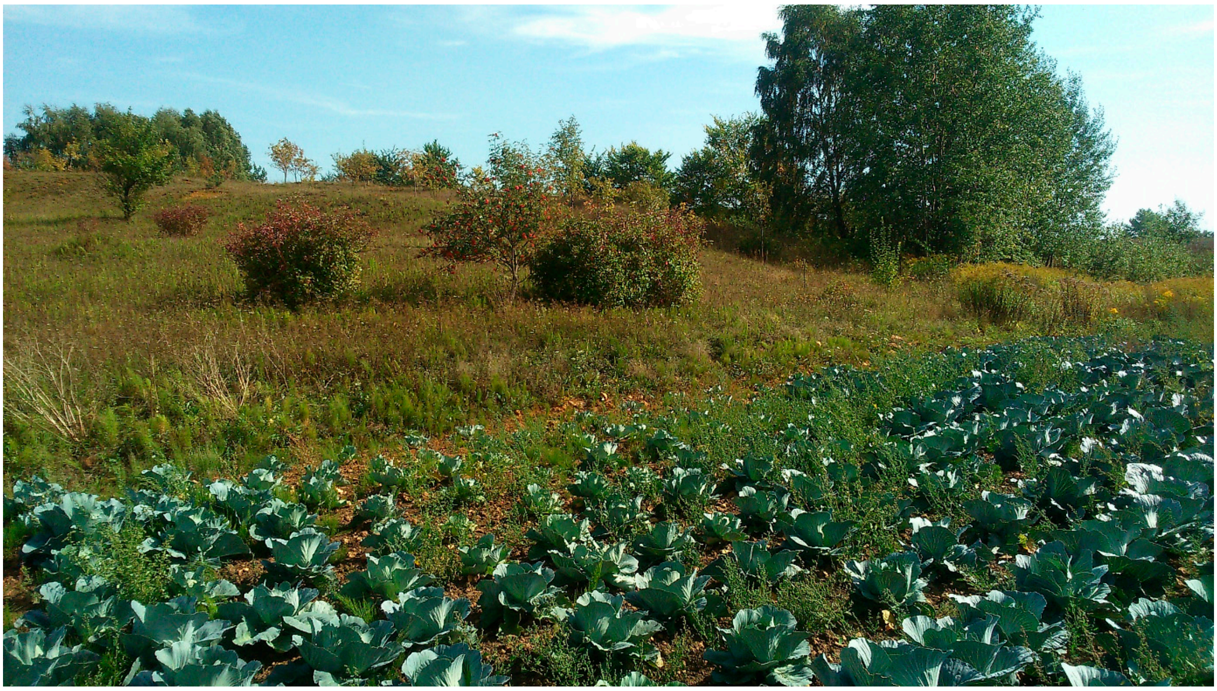

Figure S2. The border between the smelter waste pile (Dolki) and the arable land (cabbage field) - year 2018
